# Low *Aedes aegypti* Vector Competence for Zika Virus from Viremic Rhesus Macaques

**DOI:** 10.3390/v12121345

**Published:** 2020-11-24

**Authors:** Rosilainy Surubi Fernandes, Mariana Rocha David, Filipe Vieira Santos De Abreu, Anielly Ferreira-de-Brito, Noemi R. Gardinali, Sheila Maria Barbosa Lima, Márcia Cristina Ribeiro Andrade, Tatiana Kugelmeier, Jaqueline Mendes de Oliveira, Marcelo A. Pinto, Ricardo Lourenço-de-Oliveira

**Affiliations:** 1 Laboratório de Mosquitos Transmissores de Hematozoários, Instituto Oswaldo Cruz, Fiocruz, Rio de Janeiro 21040-900, Brazil; rosilainysf@gmail.com (R.S.F.); marianardavid@gmail.com (M.R.D.); filipe.vieira@ifnmg.edu.br (F.V.S.D.A.); aniellya@gmail.com (A.F.-d.-B.); 2 Laboratório de Desenvolvimento Tecnológico em Virologia, Instituto Oswaldo Cruz, Fiocruz, Rio de Janeiro 21040-900, Brazil; no_rovaris@yahoo.com.br (N.R.G.); jackie@ioc.fiocruz.br (J.M.d.O.); marcelop@ioc.fiocruz.br (M.A.P.); 3 Laboratório de Tecnologia Imunológica, Instituto de Tecnologia em Imunobiológicos Bio-Manguinhos, Fiocruz, Rio de Janeiro 21040-900, Brazil; smaria@bio.fiocruz.br; 4 Serviço de Criação de Primatas não humanos, Instituto de Ciência e Tecnologia em Biomodelos (ICTB), Fiocruz, Rio de Janeiro 21040-900, Brazil; marcia.andrade2016@gmail.com (M.C.R.A.); tkugelmeier@gmail.com (T.K.)

**Keywords:** non-human primates, rhesus macaques, Zika, viremia, vectorial capacity, transmission

## Abstract

Despite worldwide efforts to understand the transmission dynamics of Zika virus (ZIKV), scanty evaluation has been made on the vector competence of *Aedes aegypti* fed directly on viremic human and non-human primates (NHPs). We blood-fed *Ae. aegypti* from two districts in Rio de Janeiro on six ZIKV infected pregnant rhesus macaques at several time points, half of which were treated with Sofosbuvir (SOF). Mosquitoes were analyzed for vector competence after 3, 7 and 14 days of incubation. Although viremia extended up to eight days post monkey inoculation, only mosquitoes fed on the day of the peak of viremia, recorded on day two, became infected. The influence of SOF treatment could not be assessed because the drug was administered just after mosquito feeding on day two. The global infection, dissemination and transmission rates were quite low (4.09%, 1.91% and 0.54%, respectively); no mosquito was infected when viremia was below 1.26 × 10^5^ RNA copies/mL. In conclusion, *Ae. aegypti* vector competence for ZIKV from macaques is low, likely to be due to low viral load and the short duration of ZIKV viremia in primates suitable for infecting susceptible mosquitoes. If ZIKV infection in human and macaques behaves similarly, transmission of the Zika virus in nature is most strongly affected by vector density.

## 1. Introduction

Zika virus (ZIKV) was first isolated in 1947 in the Zika Forest in Uganda, from a rhesus macaque (*Macaca mulatta*) used as sentinel [1]. However, ZIKV cases were rarely reported in the following decades [2]. After an outbreak in 2007 on the western Pacific island of Yap [3], a major ZIKV epidemic was reported in French Polynesia, South Pacific, in 2013 and 2014 [4]. In 2015, the first cases of ZIKV were recorded in Brazil [5,6], and an explosive epidemic was subsequently recorded in the Americas [7]. The rapid geographical spread and the association of ZIKV infection with cases of microcephaly [8,9] and the Guillain-Barré syndrome [10,11] caused ZIKV to be declared as public health emergency in 2016, one of the most notorious infectious diseases of the decade.

In the wild (non-human) cycle, ZIKV transmission involves African non-human primates such as those from the genera *Chlorocebus* and *Cercophitecus* and several arboreal mosquitoes [2,12,13]. Natural infections in sylvatic mosquitoes were first detected in pools of *Aedes africanus* in 1948 in Uganda, but other African species have been subsequently incriminated in the transmission [1,13,14,15,16]. However outside Africa, the virus has caused severe urban and peri-urban epidemics where the domestic mosquito *Ae. aegypti* has been incriminated as the main vector [13,17,18].

Vector competence for ZIKV in *Ae. aegypti* is highly heterogeneous among different geographical origins of mosquito populations and virus lineages and isolates [19,20,21,22,23,24,25,26,27]. Technical differences between laboratory evaluations are also believed to strongly contribute to the apparent heterogeneity [13,20,27,28]. Variables such as viral titers in the oral challenge [24,29] and nature of the bloodmeal [20,30] are thought to be important factors influencing the establishment of ZIKV infection in *Ae. aegypti* and *Ae. albopictus* mosquitoes and therefore affecting vector competence [31]. The great majority of assessments of vector competence for ZIKV have used artificial bloodmeals containing thawed virus instead of feeding mosquitoes on viremic vertebrate hosts [19,20,21,25,27]. Nonetheless, it was demonstrated that an artificial bloodmeal is less infectious to *Ae. aegypti* than feeding on ZIKV viremic mice at similar viral titers [27]. However, immunocompetent non-human primates (NHP) such as rhesus macaques are the preferred translational model for ZIKV infection and pathogenesis, as well as evaluation of vaccines and preclinical interventions such as the use of antiviral drugs like sofosbuvir (SOF) during pregnancy [32,33,34,35]. Thus, to mimic ZIKV vector-borne transmission in nature, it would be preferable to feed *Ae. aegypti*—the primary epidemic ZIKV vector—directly to viremic primates in vector competence assessments. To date, however, few evaluations on the infectivity of bloodmeals on ZIKV viremic human and NHPs to *Ae. aegypti* have been performed [36,37,38]. Therefore, this work analyzes the infectivity of bloodmeals from ZIKV from two groups of viremic pregnant rhesus macaques, SOF-treated and non-treated, to two populations of *Ae. aegypti.*

## 2. Materials and Methods

### 2.1. Ethical Statement

This study was performed in compliance with the Institutional Ethics Committee on Animal Use of Instituto Oswaldo Cruz (CEUA-IOC), licenses LW-8/16 and LW-28/18 and (approved on March, 2016 and September, 2018, respectively). This study did not involve endangered or protected species.

### 2.2. Rhesus Macaques and ZIKV Infection

The study design, macaque maintenance and manipulation, viral inoculum and detection methods and drug treatment of rhesus macaques are described in detail in Gardinali et al. [35]. Briefly, the six captive-bred rhesus macaques (*Macaca mulatta*) included in this study were in the first trimester of pregnancy or early in the second trimester, clinically healthy and non-reactive in a plaque-reducing neutralization test to ZIKV, Dengue virus 1–4, Chikungunya and Yellow fever viruses. Animals were subcutaneously inoculated in the glabrous skin of the abdomen with 1 mL of supernatant of a Vero cell culture containing 10^7^ plaque-forming units (PFU)/mL of a ZIKV strain isolated from a patient from Rio de Janeiro state, Brazil, belonging to the American lineage (GenBank: KX197205). Macaques were then divided into two groups: (a) non-treated (macaques AD14, AB68 and AE62) and (b) SOF-treated (AB18, AB28, AA14) group. Sofosbuvir is an antiviral drug inhibitor of hepatitis C virus [39,40] that has proven to reduce ZIKV viral load in a mouse model [41,42] and rhesus macaques [35,41]. The treatment with SOF (5 mg SOF/Kg per day) was administered just after the mosquito feeding on the second day of post primate inoculation (d.p.p.i) in the macaques (coinciding with the peak of viremia), and interrupted at 16 d.p.p.i. The SOF was administered subcutaneously, except for macaque AB18 which was orally treated [35]. 

The macaques were housed individually in an animal facility with biological risk level 2. Blood samples were collected during the pre-inoculation period (day 0) and in the acute phase (2, 4, 8, 12, 20, 30 and 40 d.p.p.i.). Viremia was assessed with RT-qPCR in plasma samples according to Gardinali et al. [35]. Total RNA was extracted from 140 μL of plasma sample using the QIAamp Viral RNA Mini Kit (Qiagen, Hilden, Germany), according to the manufacturer’s recommendation. Quantitative RT-qPCR was performed in duplicate with 5 μL of RNA in a final reaction volume of 20 μL using TaqMan^®^ Fast Virus 1-Step Master Mix (Thermo Fisher Scientific, Waltham, MA, USA) according to the manufacturer’s protocol. Primers and probes that amplified the ZIKV membrane gene were previously described [43].

### 2.3. Mosquitoes

Two colonies of *Ae aegypti* from distinct districts in Rio de Janeiro were used: Manguinhos (≥10 generations in the laboratory) and Urca (generation F3). The eggs and larvae were incubated in an insectarium with controlled humidity, temperature and photoperiod (26 ± 1 °C, 70 ± 10% relative humidity, 12 h light cycle and 12 h dark). Larvae were reared in containers (measuring 30 × 21 × 6 cm) with one liter of dechlorinated tap water and approximately 100 larvae each, and fed with yeast (200 mg/day, LEVLIFE^®^). The adults were kept in cages under the same conditions and fed with 10% sucrose solution ad libitum until 24 h before feeding on macaques.

### 2.4. Bloodmeal on Rhesus Macaques

*Ae. aegypti* females with 5–7 days old were transferred to feeding cages containing 80 to 100 specimens each and fed directly on macaques at 1, 2, 3, 4 and 8 d.p.p.i. In two cases (macaques AB68 and AD14), batches of mosquitoes were also blood fed at 12, 18, 25 and 35 d.p.p.i. In each experiment, one feeding cage was placed directly in contact with the epidermis of the lower part of the abdomen of each macaque for 20 min during macaque anesthesia (ketamine hydrochloride at 10 mg/kg and midazolam at 0.1 mg/kg). Fully engorged mosquitoes were immediately screened on an ice bath and maintained in an incubator with controlled temperature, humidity and photoperiod (26 °C; 80% RH and with a 12 h light and 12h dark cycle). After 3, 7 and 14 days post-mosquito ingestion of bloodmeal (d.p.m.i.), batches of approximately 30 mosquitoes were submitted to forced salivation and examined as previously described [25]. Briefly, mosquito saliva was individually collected for 30 min following brief anesthesia on an ice bath and removal of the wings and legs. Then, head and body (abdomen + thorax) were separately ground in DMEM medium supplemented with 2% FBS and antibiotics/antifungals (100 units/mL of penicillin, 0.1 mg/mL of streptomycin and 0.25 µg/mL amphotericin B) and stored at −80 °C until use. The viral detection in the body, head and saliva samples was carried out both through plaque forming assay after inoculation onto confluent monolayer Vero cells and RT-qPCR according to Fernandes et al. (2020) [25]. The infection rate (IR), determined from viral detection in homogenates of mosquito body, corresponds to the percentage of infected individuals among the total number of orally challenged and examined mosquitoes. The dissemination rate (DR) was calculated from viral detection in head homogenates, corresponding to the percentage of mosquitoes with positive head among those with positive body. Finally, the transmission rate corresponds to the proportion of individuals with positive saliva among those in which the virus was able to spread to the head tissues.

### 2.5. Data Analysis

Statistical analysis was conducted in R environment [44]. Generalized linear models (GLMs) with binomial distribution, appropriate for binary data, were performed to identify significant effects of mosquito population (Manguinhos or Urca), the log of viral load (RNA copies/mL) in the macaque’s plasma at the time of mosquito feeding and the incubation time in days post-mosquito ingestion of the blood meal on macaques (3, 14 or 21 d.p.m.i.) (independent variables) on mosquito infection (dependent variable). Because positive mosquitoes were only detected among those who had fed on ZIKV infected macaques at 2 d.p.p.i., the d.p.p.i. and the treatment with SOF (which started after mosquito blood feeding at 2 d.p.p.i) were not included in GLMs. Thus, the potential effects of these variables on mosquito infection will be presented descriptively. Interactions between independent variables were also not considered in the GLM analysis due to complete separation, as ZIKV-positive mosquitoes were relatively infrequent when data was divided in many subgroups. Model selection was done through the calculation of second-order Akaike’s information criterion scores (AICs) using the “AICcmodavg” package [45]. The strength of association between each independent variable and mosquito ZIKV infection was expressed by Odds Ratio (OR) with a 95% confidence interval (95% CI). The explained deviance was calculated as: (null deviance-residual deviance)/null deviance. The influence of the same variables on virus dissemination in those mosquitoes with infected bodies (i.e., dissemination) and the presence of ZIKV in the saliva of mosquitoes with disseminated infection (i.e., transmission) could not be fitted to GLMs due to the low frequency of positive mosquitoes. Thus, dissemination and transmission data will be presented descriptively.

## 3. Results

All macaques from which mosquitoes took bloodmeal at 2, 4 and 8 d.p.p.i were confirmedly viremic, while ZIKV was not detected in any macaque plasma sample taken from 12 d.p.p.i. onwards (Table 1). Nevertheless, ZIKV infections were detected only in mosquitoes that had fed on macaques at 2 d.p.p.i. when the viral load in the macaque’s plasma peaked (1.26 × 10^5^ to 2.95 × 10^8^ RNA copies/mL for macaques AB18 and AB68, respectively; Table 1). Even so, the bloodmeals from two macaques with virus titer at 2 d.p.p.i. of 2.15 × 10^6^ and 8.13 × 10^7^ RNA copies/mL (AB28 and AD14, respectively) were not infectious to *Ae. aegypti* regardless of mosquito population and d.p.m.i.

Taking into account the total mosquitoes analyzed at 2 d.p.p.i. across all macaques they had fed on and mosquito incubation times (d.p.m.i.) (N = 366), just a small number of mosquito bodies was positive (N = 15) resulting in a global IR of 4.09%. When we analyse the results of individual macaques from which mosquitoes became infected, variation in IRs at 7 and 14 d.p.m.i were found. Considering mosquitoes fed on macaques AB68 and AE62 at 2 d.p.p.i, IRs were 6.6% and 15% at 7 d.p.m.i., and zero in mosquitos screened at 14 d.p.m.i. In mosquitoes fed on macaques AB18 and AA14, IRs were 5% and 4% at 7d.p.m.i. and 3.7% and 18% at 14 d.p.m.i (Table 1).

From the total number of infected mosquitoes, nearly half (n = 7/15) had ZIKV dissemination to head, resulting in a meager global DR of 1.91%. Dissemination was detected in only six mosquitoes examined on the 14th day after feeding AA14 and AB18, with DR ranging from 66.6–100% (Table 1). The exception was one mosquito (DR = 50%) examined at 7 days after taking a bloodmeal on macaque AE62. In total, only two mosquitoes expectorated ZIKV infected saliva at 14 d.p.m.i after feeding on a rhesus macaque (AA14). 

We investigated the effects of mosquito population, viral load in plasma of macaques at the time of mosquito blood feeding (log RNA copies/mL) and the day post mosquito ingestion of bloodmeal (d.p.m.i) on *A. aegypti* infection rates with ZIKV through GLMs with binomial distribution (Table 2).

The most informative model had macaque viral load and mosquito population as independent variables (Table 2), although three other models were also considered with high support (ΔAICc < 2, Supplementary Materials Table S1). We present results from only the most parsimonious model, since all supported GLMs had the macaque viral load as the only significant independent variable. The probability of a mosquito becoming infected with ZIKV increased with the virus titer in the macaque host (GLM OR = 1.32, OR 95% CI: 1.11–1.59, *p*-value = 0.002), with no infected mosquitoes among those fed on primates with viremia bellow 1.26 × 10^5^ RNA copies/mL for both mosquito populations (Table 1). As vector competence for arbovirus in *Ae. aegypti* may vary among geographical origin of mosquito populations, and the number of generations in laboratory may select genes modulating this phenotype [28], we used two mosquito populations of different origins and generations. However, the independent variable “mosquito population” did not show significant effect on mosquito infection, while the “d.p.m.i.” was not included in the most informative GLM, indicating no influence of these parameters on the infectivity of *Ae. aegypti* with ZIKV.

Concerning the SOF-treated group, the macaques were treated after mosquito feeding at 2 d.p.p.i., and therefore the viruses were not exposed to the drug at the time of the mosquito’s blood meal. As the few infections in mosquitoes were detected only on those fed at 2 d.p.p.i., it is difficult to suggest whether the drug has influenced or not the mosquitoes’ infectivity in the following days.

## 4. Discussion

In this study, few (4.09%) *Ae. aegypti* showed infection after feeding on ZIKV infected macaques, even when the bloodmeal was taken at the peak of viremia (2 d.p.p.i), regardless of the tested mosquito population. Moreover, the bloodmeals from two out of the six animals at their peak of ZIKV viremia were not infectious to *Ae. aegypti,* with a minimum infection threshold of 1.26 × 10^5^ RNA copies/mL. The mosquito population and the time elapsed between the infected bloodmeal and mosquito examination (d.p.m.i.) did not significantly influence the IRs, while the virus titer in the bloodmeal positively impacted the probability of a mosquito becoming infected with ZIKV. In short, the ZIKV-infected rhesus macaques developed, most of the time, viremia under the threshold to infect susceptible mosquitoes.

Our results are similar to those of Dudley et al. [38], who obtained only a single infected *Ae. aegypti* among 155 previously fed on non-pregnant rhesus macaques at the peak of viremia (ranging from 5.83  ×  10^4^ to 4.40  ×  10^6^ RNA copies/mL) of ZIKV (3 and 4 d.p.p.i.). The maximum IR found by Azar et al. [37] in *Ae. aegypti* fed on viremic non-pregnant cynomolgus macaques during the peak of viremia (7.94 × 10^6^ RNA copies/mL) was 26.2%, with half of viremic animals failing to infect any mosquito at any time point. Like Azar et al. (2018) [37], we found an increase in the infection of *Ae aegypti* mosquitoes with an increase in the viral titer in the plasma of macaques.

At the peak of viremia, reached at 2 d.p.p.i., the viral titer in macaque plasma was above 10^5^ RNA copies/mL and even reached 10^8^ RNA copies/mL, which was similar to the results found after experimental infection of ten rhesus monkeys with a 10^6^ PFU of a Thai isolate of ZIKV [46]. Interestingly, these values are higher than those observed in viremic humans in the 2007 ZIKV outbreak in Micronesia [43], the 2013–2014 outbreak in French Polynesia [47], and the 2015–2016 outbreak in Nicaragua [48]. The average viral load for humans varied from 3.5 × 10^3^ to 7.3 × 10^5^ RNA copies/mL [43,46,47] with asymptomatic presenting higher viremia titers than symptomatic patients [47]. The viremia obtained in the present study was also higher than that reported in ZIKV experimental infections in neotropical primates, whose mean viral titles did not reach 10^6^ RNA copies/mL [49,50]. As we used qRT-PCR for determining the viral load, the precise amount of infectious particles in the mosquito bloodmeals could not be evaluated, since defective RNAs were also counted. When comparing RT-PCR and lysis plate assay for Yellow Fever Virus quantification in cell culture supernatants, Bae et al. [51] found that the values obtained with such a molecular method were approximately 1000 to 5000 times higher compared to the number of infectious particles detected by lysis plate assays. Tesla et al. [52] comparing RT-qPCR and plaque assay to quantify ZIKV in *Ae. aegypti* saliva samples found that the number of infectious particles ranged from 3 to 120 PFU per sample and the RNA molecules ranged from 10^4^ to 10^7^ copies of gRNA. Thus, the number of infectious ZIKV particles in the macaques’ blood taken by the mosquitoes in our experiments could be much lower than indicated by the PCR assays. This suggests that primates usually develop low ZIKV viremia [49,50] that can barely infect *Ae. aegypti* [37,38]. Viral titer is a crucial factor in vector competence [24,53,54]. Chouin-Carneiro et al. [29] using artificial blood-feeding demonstrated that an *Ae. aegypti* mosquito population from Rio de Janeiro, as well as the ones in the present work, required a titer of at least 10^3^ PFU/mL of ZIKV.

The viremic window in which *Ae. aegypti* may become infected by feeding ZIKV infected macaques was short. The viremic peak when the few *Ae. aegypti* could get infected occurred at 2 d.p.p.i. No mosquito fed at 3 d.p.p.i. onward exhibited infection, although viremia was detected until 8 d.p.p.i. The virus titer at 8 d.p.p.i. in the blood of ZIKV injected macaques was much decreased, and no viral RNA could be detected at 12 d.p.p.i onward. Similar viral dynamics were observed in other studies, both in natural infections in humans [43] and in experimental infections in non-human primates [46,49,50]. In our study, macaques were injected with a high viral inoculum (10^7^ PFU/mL), and the animals triggered a rapid immune response [35]. Some studies have shown that the higher the dose of virus inoculated, the faster the viral infection, and concomitantly the stronger the immune response of vertebrate hosts [55,56]. Kuno and Chang [53] have emphasized the importance of length of vertebrate viremia in arbovirus transmission rates.

The precise threshold viremia that produces mosquito infection with ZIKV and other arboviruses is unknown. In addition, some factors other than virus titer in the blood of the vertebrate host may be related to mosquito infectivity. According to Azar and Weaver [30] the mosquito population, colonization history, midgut microbiota, immune system and insect-specific viruses are some of the determinants of vector competence. 

The method used to infect the vertebrate host may also influence mosquito infection with ZIKV. In addition to finding differences in pathogenesis, viral load and viremic period between rhesus macaques infected through the bite of infected mosquitoes compared to macaques needle-infected with the same virus isolate, it has been shown that mosquito infection was detected only in those fed on macaques infected by mosquito bite [30]. Dudley et al. [38], comparing two forms of ZIKA transmission in rhesus macaques, one through the bite of infected mosquitoes and the other through inoculation of the virus, observed that the infection via mosquito bite delays ZIKV replication to peak viral loads in rhesus macaques, and through deep sequencing analysis revealed that ZIKV populations in mosquito-infected macaques show greater sequence heterogeneity and lower overall diversity than in needle-inoculated animals.

In our study, all the rates we measured (IR, DR and TR) were quite low, indicating low *Ae. aegypti* vector competence for ZIKV from macaques. Indeed, when we consider the initial number of mosquitoes fed only on the peak of viremia (2 d.p.p.i), positive saliva was detected in only 0.54% (2/366), showing very low transmission efficiency. However, among those few mosquitoes that were infected, both DR and TR ranged from 50 to 100%. This data suggests that, once infected, the chances of ZIKV spreading to secondary tissues and being transmitted by *Ae. aegypti* are high, mainly if an elevated viral replication in the mosquito gut is achieved [19,25,31].

Studies of *Ae. aegypti* vector competence for ZIKV from primates are rare and usually use old laboratory strains of mosquito, or test a small number of mosquitoes with viral isolates not from the same place as the mosquitoes were originally collected [30,38]. The present study gathers results from numerous tests carried out with a combination of virus and mosquito population from the same geographical origin with the goal of mimicking natural ZIKV vector-borne transmission.

The virus strain we used was isolated in 2015 from a patient from Rio de Janeiro, Southeast Brazil, and belongs to the American lineage. Various contemporaneous ZIKV isolates from Rio de Janeiro as well as from other sites in Brazil have proved to be very infective to *Ae. aegypti* populations used in the present work. Indeed, when challenged with artificial bloodmeals containing ≥10^6^ PFU of ZIKV/mL, like other assessments of vector competence [19,20,21,22,23,24,25,26,27], the Urca *Ae. aegypti* has exhibited very high IR, DR and TR [21,29,57]. In addition, when the Rio de Janeiro *Ae. aegypti* population (whose gene pool included Urca) was orally challenged with ZIKV isolates from the American and Asian lineages, mosquitoes showed higher vector competence for the American lineage compared to the Asian lineage [25] Therefore, the viral strain and the mosquito populations used herein were not responsible for the described low vector competence of *Ae. aegypti* from viremic rhesus macaques, but the low viral load and short duration of ZIKV viremia in the primates. Thus, our conclusion of low *Ae. aegypti* vector competence for ZIKV from primates is not limited to a particular ZIKV isolate and mosquito population, as it is consistent with the two previous studies using macaques infected with distinct ZIKV and mosquito strains. [37,38].

Is *Ae. aegypti* vector competence for ZIKV obtained from primates usually as low as our and previous data on macaques and humans have shown? If so, it would be hard to explain the recent explosive ZIKV epidemic in Brazil and other American countries based only on vector competence experimental results. However, vector competence is one of the multiple components influencing vectorial capacity, including vector density in relation to the host, bite rate, mosquito female longevity and extrinsic incubation period (EIP) [54,58,59]. Thus, despite the low vector competence from viremic primates described here and in previous works [37,38], *Ae. aegypti* is unquestionably the ZIKV primary vector [13,17,18]. A poorly competent vector can, however, sustain or enlarge an outbreak if the EIP is brief, if bites on hosts are frequent or if the vector population density is high. If humans are similar to macaques in terms of viremia duration and viral load in blood and infectivity for *Ae. aegypti*, our results together with previous findings suggest that the dynamics of Zika transmission in nature is strongly influenced by the population density of vectors. A large number of daily mosquito bites are certainly required to assure some are on hosts at the brief time of maximum viral load in blood, and a small fraction may become infected in order to trigger and sustain vector transmission. Therefore, reducing *Ae. aegypti* population density is decisive in mitigating ZIKV transmission.

## Figures and Tables

**Table 1:** Infection, Dissemination and Transmission Rates of *Aedes aegypti* mosquitoes blood-fed on Zika virus (ZIKV) inoculated pregnant rhesus monkeys.

Monkey Identification (Treatment)	Mosquito Population	d.p.p.i. ^a^ Monkey (RNA Copies/mL of Plasma)	d.p.m.i. ^b^ Mosquitoes	N ^c^	Infection Rate ^d^ (Number of Positive Bodies)	Disseminated Rate ^e^ (Number of Positive Heads)	Transmission Rate ^f^ (Number of Positive Salivas)
AB68 (non-treated)	*Aedes aegypti* Manguinhos F > 10	2 (2.95 × 10^8^)	3	20	5% (1)	0%	0%
			7	20	15% (3)	0%	0%
			14	12	0%	0%	0%
		4 (3.02 × 10^2^)	3	15	0%	0%	0%
			7	20	0%	0%	0%
			14	20	0%	0%	0%
		8 (1.32 × 10^6^)	3	8	0%	0%	0%
			7	20	0%	0%	0%
			14	18	0%	0%	0%
		12 (0)	14	2	0%	0%	0%
		18 (0)	3	20	0%	0%	0%
			14	20	0%	0%	0%
		25 (0)	3	20	0%	0%	0%
			14	20	0%	0%	0%
		35 (0)	7	20	0%	0%	0%
AD14 (non-treated)	*Aedes aegypti* Manguinhos F > 10	2 (8.13 × 10^7^)	3	20	0%	0%	0%
			7	20	0%	0%	0%
			14	4	0%	0%	0%
		4 (5.01 × 10^4^)	3	9	0%	0%	0%
			7	20	0%	0%	0%
			14	20	0%	0%	0%
		8 (9.12 × 10^4^)	3	8	0%	0%	0%
			7	20	0%	0%	0%
			14	14	0%	0%	0%
		12 (0)	3	20	0%	0%	0%
			7	20	0%	0%	0%
			14	20	0%	0%	0%
		18 (0)	3	20	0%	0%	0%
			7	20	0%	0%	0%
			14	20	0%	0%	0%
		25 (0)	3	20	0%	0%	0%
			7	20	0%	0%	0%
			14	20	0%	0%	0%
		35 (0)	7	20	0%	0%	0%
AE62 (non-treated)	*Aedes aegypti* Manguinhos F > 10	2 (1.20 × 10^7^)	7	30	6.6% (2)	50% (1)	0%
			14	30	0%	0%	0%
		4 (2.69 × 10^4^)	7	30	0%	0%	0%
			14	23	0%	0%	0%
AB18 (SOF-treated) ^g^	*Aedes aegypti* *Manguinhos* F > 10	2 (1.26 × 10^5^)	7	20	5% (1)	0%	0%
			14	30	6.6% (2)	100% (2)	--
		4 (1.0 × 10^5^)	7	20	0%	0%	0%
			14	30	0%	0%	0%
		8 (1.0 × 10^3^)	7	20	0%	0%	0%
			14	30	0%	0%	0%
AB18 (SOF-treated) ^g^	*Aedes aegypti* Urca F3	2 (1.26 × 10^5^)	7	15	0%	0%	0%
			14	11	18.1% (1)	100% (1)	--
		4 (1.0 × 10^5^)	7	20	0%	0%	0%
			14	25	0%	0%	0%
		8 (1.0 × 10^3^)	7	20	0%	0%	0%
			14	30	0%	0%	0%
AA14 (SOF-treated) ^g^	*Aedes aegypti* *Manguinhos* F > 10	1 (NA)	14	19	0%	0%	0%
		2 (2.8 × 10^6^)	7	25	0%	0%	0%
			14	27	3.7% (1)	100% (1)	100% (1)
		3 (NA)	14	23	0%	0%	0%
AA14 (SOF-treated) ^g^	*Aedes aegypti* Urca	2 (2.8 × 10^6^)	7	30	4% (1)	0%	0%
			14	30	10% (3)	66.6% (2)	50% (1)
		3 (NA)	14	20	0%	0%	0%
AB28 (SOF-treated) ^g^	*Aedes aegypti* *Manguinhos* F > 10	2 (2.15 × 10^6^)	14	5	0%	0%	0%
AB28 (SOF-treated) ^g^	*Aedes aegypti* Urca	1 (NA)	14	1	0%	0%	0%
		2 (2.15 × 10^6^)	14	17	0%	0%	0%
		3 (NA)	14	5	0%	0%	0%

^a^. days post-primate infection (d.p.p.i.) with ZIKV when mosquito took the bloodmeal from rhesus monkey; ^b^. days post-mosquito ingestion (d.p.m.i.) of the bloodmeal on rhesus monkey; ^c^. Number of mosquitoes tested per d.p.m.i.; ^d^. Infection rate (IR) refers to the proportion of mosquitoes with infected body (abdomen and thorax) among tested mosquitoes; ^e^. Disseminated infection rate (DR) corresponds to the proportion of mosquitoes with infected head among infected mosquitoes (i.e., abdomen/thorax positive); ^f^. Transmission rate (TR) refers to the proportion of mosquitoes with infected saliva among mosquito with disseminated infection (i.e., positive head); ^g^: daily Sofosbuvir (SOF) treatment was initiated after the mosquito were blood fed at 2 d.p.p.i; DR and TR were determined both by plaque forming assay and RT-qPCR; NA: viral load in plasma not analyzed.

**Table 2:** Generalized linear models (GLMs) with binomial distribution showing the influence of macaque viral load (log) on *Aedes aegypti* Zika virus infection.

GLM: Infection ~ log (Macaque Viral load) + Mosquito Population						
Independent Variable	Coefficient (β)	Std. Error	OR (95% CI)	Z Value	*p*-Value	Explained Deviance
Intercept	−8.26	1.53		−5.39	<0.001	8.18%
Macaque viral load (log)	0.28	0.09	1.33 (1.11–1.59)	3.09	0.002	
Mosquito population (Urca)	1.01	0.61	2.73 (0.82–9.11)	1.640	0.101	

CI: Confidence interval; OR: odds ratio; 95% CI: 95% confidence interval; Std. error: standard error.

## Supplementary Materials

The following are available online at https://www.mdpi.com/1999-4915/12/12/1345/s1, Table S1: Competitive generalized linear models of the Aedes aegypti infection with Zika virus after feeding on infected rhesus macaques.
