# Supplementary material for: Low Aedes aegypti Vector Competence for Zika Virus from Viremic Rhesus Macaques
Source: Viruses. 2020 Nov 24;12(12):1345. doi: 10.3390/v12121345 (PMC7759330; doi:10.3390/v12121345)
Supplement: Supplementary file 1 [file viruses-12-01345-s001.pdf]

S1 Table. Competitive generalized linear models of the *Aedes aegypti* infection with Zika virus after feeding on infected rhesus macaques.

Model	K	AICc	ΔAICc	ModelLik	AICcWt	LL
Null model	1	153.59	14.62	0	0	-75.79
Infection ~ mosquito population	2	152.54	9.58	0.01	0	-74.26
**Infection ~ log (macaque viral load)**	**2**	**143.46**	**0.5**	**0.78**	**0.24**	**-69.72**
Infection ~ d.p.m.i.	2	153.12	10.15	0.01	0	-74.55
**Infection ~ mosquito population + log (macaque viral load)**	**3**	**142.96**	**0**	**1**	**0.31**	**-68.47**
Infection ~ mosquito population + d.p.m.i.	3	154.56	11.59	0	0	-74.26
**Infection ~ log (macaque viral load) + d.p.m.i.**	**3**	**144.5**	**1.54**	**0.46**	**0.14**	**-69.24**
**Infection ~ mosquito population + log (macaque viral load) + d.p.m.i.**	**4**	**144.42**	**1.45**	**0.48**	**0.15**	**-68.18**

d.p.m.i.: days post mosquito infection; K: number of parameters; AICc: Akaike Information Criterion corrected; ΔAICc: difference between the AICc of a given model and the lowest AICc, which ΔAICc > 2 means high support for a given model; Wt: model probability based on Akaike weight. Best GLMs are in bold.
